# Specificity of Genetic Biomarker Studies in Cancer Research: A Systematic Review

**DOI:** 10.1371/journal.pone.0156489

**Published:** 2016-07-06

**Authors:** Garrett Green, Ruben Carmona, Kaveh Zakeri, Chih-Han Lee, Saif Borgan, Zaid Marhoon, Andrew Sharabi, Loren K. Mell

**Affiliations:** Moores Cancer Center, University of California San Diego, La Jolla, California, United States of America; Catalan Institute of Oncology, SPAIN

## Abstract

As genetic information becomes more readily available, there is increasing demand from both patients and providers to develop personalized approaches to cancer care. Investigators are increasingly reporting numbers of studies correlating genomic signatures and other biomarkers to survival endpoints. The extent to which cancer-specific and non-specific effects are reported in contemporary studies is unknown. In this review of 85 high-impact studies associating genetic biomarkers with cancer outcomes, 95% reported significant associations with event-free survival outcomes, yet less than half reported effects on a cancer-specific endpoint. This methodology leaves open the possibility that observed associations are unrelated to cancer.

## Introduction

### Prognostic Biomarker In Clinical Practice

Gene expression diagnostics and related biomarkers are useful for risk-stratifying patients according to their potential to benefit from various treatment approaches [1]. Compared to conventional clinical and pathologic criteria, biomarkers have augmented the prognostic and predictive information available to patients. For example, Oncotype DX, a commercially available gene signature, helps predict which patients with node-negative breast cancer will benefit from adjuvant chemotherapy [2]. Ideally, correlating the gene expression profiles with outcomes will lead to improved cancer outcomes.

The U.S. National Cancer Institute has increasingly emphasized biomarker development, with the goal “to deliver the right drug to the right patient at the right time” [3]. For example, clinical trials such as the NCI-Molecular Analysis for Therapy Choice (NCI-MATCH) will analyze patients’ tumors to determine whether they contain genetic abnormalities with an actionable drug target and assign treatment based on the abnormality. Similarly the first ever American Society of Clinical Oncology (ASCO) Targeted Agent and Profiling Utilization Registry (TAPUR) study is a prospective non-randomized clinical trial that will deliver specific anticancer drugs based upon identified genomic variations in a patient’s tumor. These studies as well as the developing field of cell-free tumor DNA or “liquid biopsies” highlight the critical role biomarkers will play in the future of oncology.

Over the past decade an increasing number of studies associating gene expression profiles with event-free survival outcomes have been reported. However, in patients at risk for competing causes of mortality, associations between biomarker and poorer survival may be unrelated to cancer. This could result in overtreatment, by combining patients at high risk of cancer mortality and those at risk of mortality from other causes into the same risk pool. To determine optimal treatment strategies, it is necessary to distinguish whether cancer or non-cancer events are related to the biomarker effect.

### How Genetic Biomarkers Predict Clinical Outcomes

Various methods for validating gene signatures have been used. Frequently researchers perform RNA-based analysis of formalin fixed paraffin embedded cancer cells to identify genes that are relatively over- or under-expressed. A common approach to biomarker discovery is the “top down” approach, where a set of known clinical outcomes is correlated with characteristic gene expression patterns without any biological assumptions specified *a priori* [4,5]. In contrast, the “bottom up” approach involves identifying gene expression profiles linked to a specific biological process (such as metastasis, invasion, cell cycle regulation, angiogenesis, etc.) with poorer outcomes or features known to be associated with poor outcomes (such as high grade) [5].

While the methodology behind the validation of genetic biomarkers is well-developed, the technique does not require that the mechanism of the gene products or their relationship with outcomes be understood, yet inferences would differ considerably depending on whether the expression profile was correlated with cancer-specific or non-specific events. The importance of reporting effects of treatments and other covariates on both cancer-specific endpoints, such as cancer recurrence or mortality, and competing events, such as non-cancer mortality, is well established in the clinical medical literature [6]. However, it is not clear how well this knowledge has been disseminated amongst the basic research community.

Many cancer patients are at high risk of competing causes of death unrelated to cancer, for example due to age or underlying cardiovascular or pulmonary comorbidities. When only effects on combined endpoints such as overall survival (which aggregates cancer and non-cancer mortality) or disease-free survival (which typically aggregates cancer recurrence and death from any cause) are reported, it is possible that an effect could correspond in whole or part to the non-cancer part of the endpoint, which would have a significant impact on the inferences of the effect with respect to cancer. It is known that even amongst randomized trials published in leading medical journals, investigators frequently neglect to report cause-specific effects [6]. We hypothesized that a similar problem would affect studies correlating genetic biomarkers with clinical outcomes, and sought to interrogate this question through a systematic literature review.

## Materials and Methods

### Outcomes Reporting In Genetic Biomarker Studies

**Fig 1:**
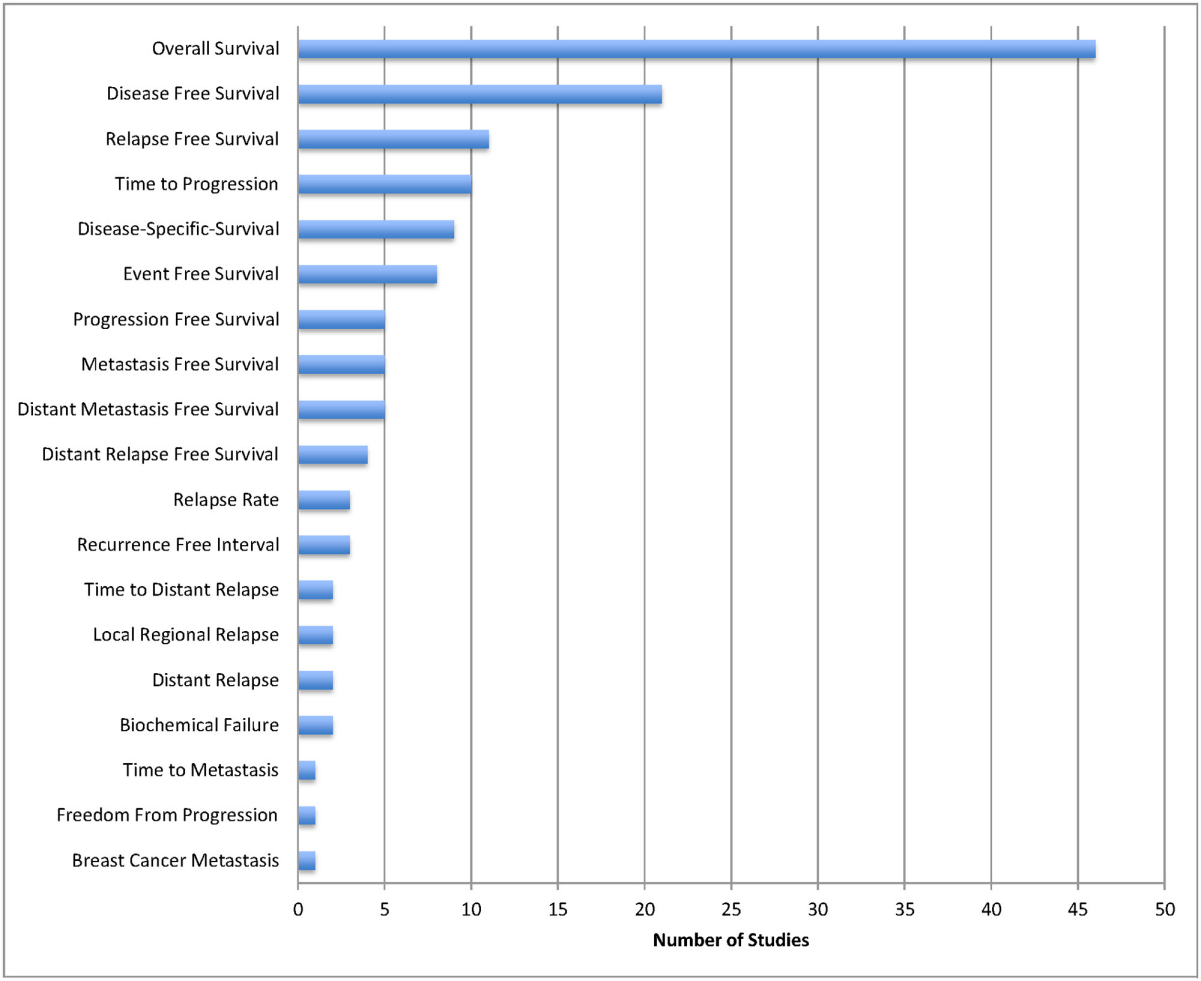
Flow diagram of exclusion criteria.

Our primary aim was to estimate the proportion of contemporary studies OF genetic biomarkers in oncology that report their effects on both cancer-specific and competing events. Secondary aims were to estimate how often a primary endpoint could be identified, how often outcomes were defined, and how many articles reported statistically significant effects on clinical outcomes. The study design was a systematic review, based on methods defined *a priori* and implemented previously [6] (Fig 1 and S1 File). We followed the PRISMA guidelines (S3 File) for reporting results of systematic reviews [7]. We searched MEDLINE for studies published between January 1, 2007 and August 1, 2014 analyzing overall survival or at least one other event-free survival (EFS) endpoint (defined as an endpoint combining one or more cancer-specific events with death from any cause). This time period was chosen to represent contemporary articles indicative of prevailing reporting norms and guidelines, and to be wide enough to yield a representative sample while narrow enough to yield a manageable set of articles for detailed review. Examples of biomarkers we analyzed were multigene expression signatures such as CINSARC sarcoma and leukemia stem cell specific gene signatures.

We selected studies from 10 journals with high 5-year impact factor in 2013 (JNCI, JAMA, NEJM, Lancet, Nature, JCO, PNAS, Cancer Research, Nature Medicine, Nature Genetics) [8], yielding 253 studies for further review (S2 File). We selected these journals to represent a high level of reporting standards in the medical literature. We excluded preclinical studies (n = 76), commentaries (n = 10), meta-analyses or reviews (n = 6), studies involving multiple cancers (n = 4), studies lacking time to event data (n = 5), studies unavailable online, and studies exclusively in metastatic disease (n = 66), leaving 85 studies for analysis (Fig 1). Metastatic disease studies were excluded because competing non-cancer events were expected to be low.

All studies were reviewed and the following data were extracted: disease site, primary (and secondary, if reported) endpoint(s)—if identified, endpoint definition(s)–if identified, and results of tests of statistical significance. If an EFS endpoint was not explicitly defined, we assumed that the authors followed common conventions [9] and included death from any cause as an event, while endpoints that referred exclusively to events such as recurrence, metastasis, locoregional control, etc. were cause-specific. Articles were categorized according to whether effects on both cancer-specific and non-cancer events were reported, what statistical analyses (if any) were performed, and whether clinicopathologic associations were reported. Wilson’s method [10] was used to estimate the 95% confidence interval (CI) for our primary endpoint. Fisher’s exact test was used to test differences according to disease site.

## Results

**Fig 2:**
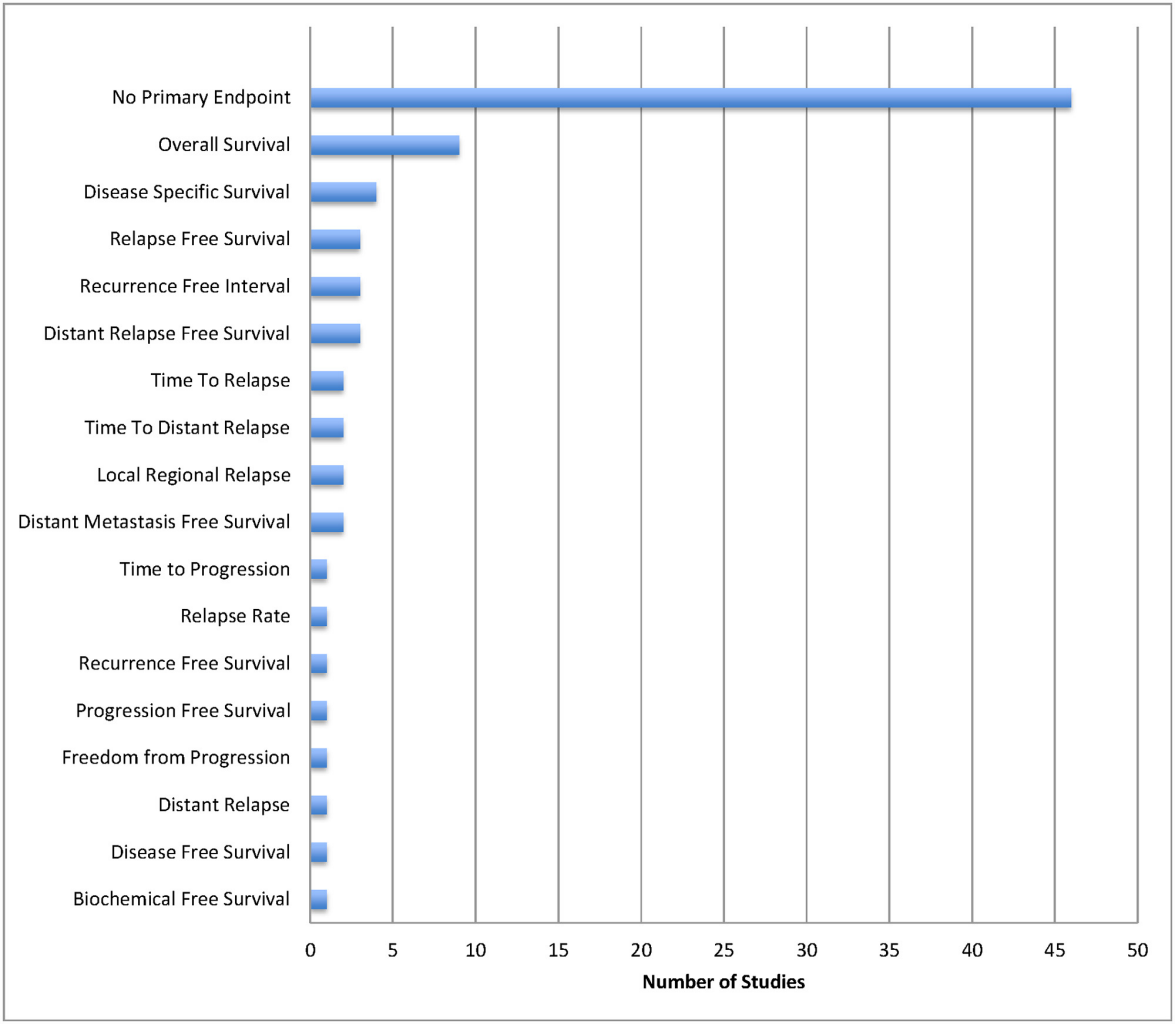
Histogram of (A) All Endpoints and (B) Primary Endpoints Reported.

The majority of studies included were in breast cancer (27%) and leukemia/lymphoma (25%). We found that 81 studies (95%) reported a statistically significant association with at least one clinical outcome. The most common endpoints reported were overall survival (33%) and disease-free survival (15%) (Fig 2). 46 studies (54%) did not identify a primary outcome or endpoint (Fig 2), and 28 studies (33%) did not define the endpoint(s) that was (were) being reported.

Overall, we found that 54% of studies (95% confidence interval (CI), 44%-64%) did not report effects of the biomarker on any cancer-specific outcome. However, 83 studies (98%) did report associations with clinicopathologic cancer-specific factors, such as stage or grade. We observed that studies in genitourinary cancer (p < 0.05) were significantly more likely to report effects on a cancer-specific outcome compared to other disease sites.

In summary, a high proportion of studies purporting to show significant associations between gene expression and clinical outcomes did not define or identify the primary endpoint of interest, or report effects on a cause-specific outcome. This occurred despite restricting our analysis to studies published in highly selective journals.

### Recommendations

Pitfalls in using EFS endpoints, including overall survival, have been frequently discussed in the medical literature [11–15]. Confounding by non-specificity is an important problem that can undermine the validity of conclusions from clinical studies, including population-based analyses and randomized trials [16–18]. This form of confounding may contribute to publication bias as well (by way of confirmation bias), a problem known to beset scientific literature [19]. Such bias can occur when investigators observe the positive effect of a treatment on survival they hoped to find, despite the effect being wholly or partially attributable to positive effects on non-specific events (such as non-cancer mortality). This effect may be traced to either selection bias or random imbalances in unmeasured factors [16,17]. Interestingly, a remarkably high percentage of studies in our sample (95%) were “positive” (i.e., reported statistically significant associations between their biomarker and a clinical outcome).

For studies reporting associations between outcomes and biomarkers, we recommend the following steps, in keeping with guidelines promulgated by other investigators [20]:

1. Clearly identify the study’s primary endpoint(s) (i.e., the endpoint or set of endpoints used for sample size (or power) calculation), and secondary endpoint(s), if any.
2. Clearly identify the starting point for time-to-event calculations (e.g., date of registration, date of diagnosis, date of treatment completion, etc.)
3. For composite endpoints, clearly identify the events comprising the endpoint and criteria used for censoring. In particular, investigators should indicate whether “death from any cause” is treated as an event. Endpoints termed “progression”, “recurrence”, “failure”, “time to progression”, “time to recurrence”, “time to failure”, “distant metastasis”, “local control” or “locoregional control”, and “cause-specific mortality” or “cancer mortality” are cause-specific and should treat deaths from competing causes as censored, whereas endpoints termed “progression-free survival”, “disease-free survival”, “event-free survival”, etc. are not cause-specific, and should treat death from any cause as an event.
4. Define the protocol used for assessing time to recurrence/progression, including frequency of clinic visits and imaging, type of imaging used, whether biopsy was required, and indications used to trigger visits, imaging, or biopsy.
5. Clearly and separately distinguish effects on cause-specific events from effects on non-specific or competing events (particularly competing mortality), along with appropriate tests of statistical significance
6. Clearly identify the statistical methods and/or models used to test associations, including criteria for significance, how covariates were coded and controlled, how assumptions of the models were checked, and criteria for including/excluding covariates from the model

A nice example from the literature we reviewed, which we recommend emulating, was the study by Yothers et al. [21].

## Conclusions

Specificity is as crucial in outcomes research as any branch of science. Our findings indicate that a high proportion of studies in oncology analyzing associations between gene expression biomarkers and clinical outcomes use non-specific methodology. We restricted our analysis to high impact journals so the extent of this problem in likely even greater in the broader medical literature. Our sample omitted studies in metastatic disease and was temporally restricted to more recent studies, but we expected the quality of reporting would be higher for studies in competing risks settings, where non-specificity is of greater concern, and in modern articles, due to more recent publication of reporting guidelines. We recommend that such studies place greater attention on reporting both cancer-specific and non-specific effects to facilitate their interpretation.

## Supporting Information

S1 File Protocol. (DOCX)

S2 File Pubmed Identification Numbers for 85 studies included in the analysis. (DOCX)

S3 File Prisma Checklist. (DOC)
