# Supplementary material for: Specificity of Genetic Biomarker Studies in Cancer Research: A Systematic Review
Source: PLoS One. 2016 Jul 6;11(7):e0156489. doi: 10.1371/journal.pone.0156489 (PMC4934683; doi:10.1371/journal.pone.0156489)
Supplement: S1 File — (DOCX) [file pone.0156489.s001.docx]

**S1 File.** Protocol

1. Background: Composite primary endpoints are widely used in tumor gene expression biomarker studies. Their usage implicitly assumes a homogeneous treatment effects on both cancer and non- cancer events. When this assumption does not hold, the interpretation of study results can be obscured and accurate sample size estimation is impeded. The extent to which effects on primary cancer and non-cancer events are analyzed separately in contemporary biomarker studies is unknown. The purpose of this study is to determine the extent to which cause-specific effects on primary events are reported in tumor gene expression biomarker publications.

2. Search Strategy: We will perform a MEDLINE search of ten leading medical journals (JAMA, NEJM, Lancet, Nature, JCO, JNCI, PNAS, Cancer Research, Nature Medicine, Nature Genetics) between between January 1, 2007 and August 1, 2014.

(cancer[Title] OR leukemia[Title] OR lymphoma[Title] OR carcinoma[Title] OR melanoma[Title] OR sarcoma[Title] OR tumor[Title] OR tumour[Title]) AND

(survival[Title] OR outcome[Title] OR response[Title] OR relapse[Title] OR recurrence[Title] OR death [Title] OR progression[Title] OR prognosis[Title]) AND

(overexpression[Title] OR expression[Title] OR profiling[Title] OR signature[Title] OR profile[Title] OR gene[Title] OR genetic[Title] OR multigene[Title] OR genomic[Title] OR proteogenomic[Title] OR amplification[Title] OR amplified[Title]) AND

("2007/01/01"[PDAT] : "2014/08/01"[PDAT])

The time period is chosen to represent contemporary articles indicative of prevailing reporting norms and guidelines. The time window is selected to be wide enough to yield a representative sample and narrow enough to yield a manageable set of articles for detailed review.

4. Selection Criteria

The population of interest is the set of published gene expression biomarker studies. Inclusion criteria are studies in non-metastatic/non-recurrent cancer that use a primary disease specific survival endpoint. A “disease-specific survival” (DSS) endpoint is defined as being comprised of only cancer-specific events (e.g., recurrence, cancer death, etc.) and death from cancer. Exclusion criteria include articles reporting animal studies, secondary analyses, multiple cancers, commentaries, prevention studies. The sample will be drawn from the set of articles published within past 7 years in ten leading medical journals in oncology.

Studies were reviewed and data extracted included disease site, primary endpoint, endpoint definition, and statistical significance from each article. Articles will be categorized according to whether cancer specific effects were reported or if a composite endpoint was reported. Articles will also be categorized according to whether a statistical analysis of the effect of the gene expression on the endpoint was performed. Fisher exact test will be used to test differences in categorization according to disease site.

5. Study Quality Assessment Studies are selected from contemporary articles published in leading medical journals, in order to be representative of the highest standards attained in the medical literature

6. Data Extraction Studies will be initially screened for inclusion / exclusion criteria: (1) recurrent/metastatic disease (2) secondary analysis (3) prevention study (4) non-clinical study defined (5) study was retracted. For each article meeting inclusion criteria, the following data will be abstracted: (1) article Pubmed ID (2) journal name (3) disease site (4) primary endpoint used (5) endpoint definition (6) whether statistical analysis of effect of biomarker on endpoint was reported (7) whether clinicopathologic information was collected.

7. Synthesis: Analysis will be in the form of tables.

8. Study Limitations: There are no conflicts of interest. The study has a potentially biased sample by restricting to only 10 journals. However, these are among the most respected journals in oncology, so our assumption is that the direction of bias is in favor of more high quality reporting in these journals. Therefore if we find limitations in reporting we assume this is not likely significantly better in journals we did not analyze. There is a temporal bias in our sample but by the same rationale, we expect the quality of reporting to be highest in modern articles due to the accumulation of knowledge and publication of guidelines for reporting in the modern era.

9. Reporting: This study has implications for all clinical investigators, particularly those involved in cancer clinical trials and their design. Persons involved in study design, conduct, and analysis will be considered authors of publications resulting from this work.

10. Schedule: 2014 – Data collection and analysis 2014 – Preparation and submission of findings for publication

11. This study was not funded.
