# Supplementary material for: Specificity of Genetic Biomarker Studies in Cancer Research: A Systematic Review
Source: PLoS One. 2016 Jul 6;11(7):e0156489. doi: 10.1371/journal.pone.0156489 (PMC4934683; doi:10.1371/journal.pone.0156489)
Supplement: S2 File — (DOCX) [file pone.0156489.s002.docx]

**Supporting Information S2 File.** Pubmed Identification Numbers for 85 studies included in the analysis

**
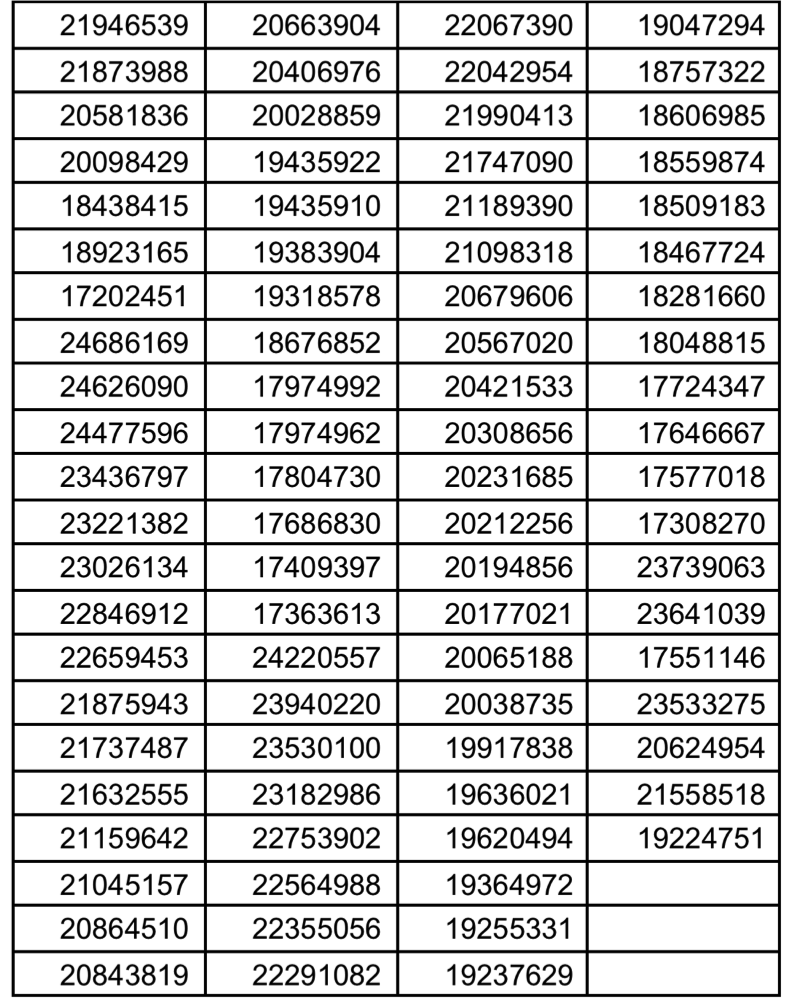
**
